# A New Measure for Assessing the Intensity of Addiction Memory in Illicit Drug Users: The Addiction Memory Intensity Scale

**DOI:** 10.3390/jcm7120467

**Published:** 2018-11-22

**Authors:** Jia-yan Chen, Jie-pin Cao, Yun-cui Wang, Shuai-qi Li, Zeng-zhen Wang

**Affiliations:** 1 Department of Epidemiology and Biostatistics, School of Public Health, Tongji Medical College, Huazhong University of Science and Technology, Wuhan 430030, China; palachen@hust.edu.cn (J.-y.C.); wangyuncui2017@163.com (Y.-c.W.); omni_slash@163.com (S.-q.L.); 2 Tongji Research Centre of Mental Health, Huazhong University of Science and Technology, Wuhan 430030, China; jiepin.cao@duke.edu; 3 School of Nursing, Duke University, Durham, NC 27710, USA; 4 School of Nursing, Hubei University of Chinese Medicine, Wuhan 430065, China

**Keywords:** addiction, memory, assessment, substance use disorder

## Abstract

Disrupting the process of memory reconsolidation could be a promising treatment for addiction. However, its application may be constrained by the intensity of addiction memory. This study aimed to develop and initially validate a new measure, the Addiction Memory Intensity Scale (AMIS), for assessing the intensity of addiction memory in illicit drug users. Two studies were conducted in China for item analysis (*n* = 345) and initial validation (*n* = 1550) of the AMIS. The nine-item AMIS was found to have two factors (labelled Visual Clarity and Other Sensory Intensity), which accounted for 64.11% of the total variance. The two-factor structure provided a reasonable fit for sample data and was invariant across groups of different genders and different primary drugs of use. Significant correlations were found between scores on the AMIS and the measures of craving. The AMIS and its factors showed good internal consistency (Cronbach’s *α*: 0.72–0.89) and test-retest reliability (*r*: 0.72–0.80). These results suggest that the AMIS, which demonstrates an advantage as it is brief and easy to administer, is a reliable and valid tool for measuring the intensity of addiction memory in illicit drug users, and has the potential to be useful in future clinical research.

## 1. Introduction

Addiction memory is conceptualized as a pathological memory related to addictive behaviors [1,2]. Even after long-term abstinence, addiction memory can be reactivated upon re-exposure to substance-related cues and associated with a craving that results in relapse [2,3]. Recently, several studies have found that disrupting the reconsolidation of drug-related memory can reduce craving, attention bias, or drug-using behavior [4,5,6,7], indicating that addiction memory can be manipulated during reconsolidation. However, several “boundary conditions” may constrain the application of memory reconsolidation in addiction treatment [8,9]. One of the constraints is the intensity of memory. It has been shown that memories with a higher level of intensity may be more resistant to disruption and require a longer re-exposure (or a shorter time interval between memory acquisition and re-exposure) to induce memory lability [10,11,12]. Given that, a valid measurement for assessing the intensity of addiction memory is needed before examining the impact of memory intensity on reconsolidation propensity.

Previously, a single-item visual analogue scale (VAS) was used in some studies to assess the vividness of addiction memory [13,14]. Although the VAS has been widely used to evaluate the subjective experience, such as craving [15], this single-item measure may fail to reflect the complex nature of the intensity of addiction memory. Regarding instruments measuring the intensity of addiction memory, we believe that the use of a multi-item scale or questionnaire would be beneficial for research in that it could help to clarify the underlying dimensions [16]. Furthermore, although there have been several scales and questionnaires developed to assess the severity of addiction [17,18,19,20,21], we should note that these instruments were not originally designed to measure the intensity of addiction memory. It is unknown which items or dimensions of the tools may reflect the memory strength, which raises concern about the utility of these indexes in assessing the intensity of addiction memory. Given these concerns, a new measure is needed to evaluate the intensity of addiction memory explicitly.

Studies on the phenomenology of memory, which mainly focus on one’s subjective experience retrieved from the memory [22,23], open an opportunity to assess the intensity of addiction memory. Although little is known about the phenomenological characteristics of addiction memory, previous studies have suggested a relationship between addiction memory and autobiographical memory, especially episodic memory [2,3,24]. Similar to the autobiographical memory, addiction memory is related to personal experiences derived from an individual’s history of drug use. Thus, research on autobiographical memory can be used as a frame of reference. There have been several studies presenting a diverse variety of phenomenological characteristics of autobiographical memory, such as vividness and sensory details [22,25,26,27,28,29,30]. Although the classification of phenomenological traits is still under discussion, the characteristics involving the sensory-perceptual information could probably be used as a central measure of memory intensity. The sensory-perceptual details are the primary information retrieved from one’s autobiographical (episodic) memories [31]. In the self-memory system, the sensory-perceptual information of autobiographical memory can be directly retrieved when there are event-related cues [31], meaning that the sensory details would be re-experienced when recalling the memory. In that, people may feel a high level of sensory intensity if they retrieve detailed sensory information. Additionally, the visual imagery is presented predominately and correlated with other sensory details (e.g., olfactory, gustatory) in episodic information [32,33]. In sum, a strong memory tends to be visually vivid, full of sensory details. Since the addiction memory is characterized by powerful imprints of the information about psychoactive substances [2,3,24], we infer that the strength of sensory-perceptual information could be used for measuring the intensity of addiction memory.

Although the studies on autobiographical memory provide a research frame for understanding the properties of addiction memory, there is one crucial limitation when applying the measurements of autobiographical memory in assessing the intensity of addiction memory. It should be emphasized that the measures were developed to evaluate the autobiographical memories of individuals about their life events but not specifically their addiction memories. To the best of our knowledge, there is no scale or questionnaire specifically designed for assessing the phenomenological characteristics in the strength of addiction memory. Therefore, our study aimed to fill this gap first through the development and initial validation of a scale specifically for evaluating the intensity of addiction memory in illicit drug users. In our research, a Likert-type scale, which we labelled the Addiction Memory Intensity Scale (AMIS), was initially developed. We conducted two studies: one for item analysis and one for initial validation of the AMIS. We hypothesized that the scale would be a reliable and valid tool for assessing the intensity of addiction memory in illicit drug users. 

## 2. Materials and Methods

### 2.1. Initial Scale Development

The development of the AMIS began with a review of the literature on the phenomenology of autobiographical memory. Instruments that measured the phenomenological characteristics of autobiographical memory [22,25,26,27,28,29,30], especially the items involving sensory-perceptual information, were collected as the reference. Regarding the domains of memory intensity, we agree with the previous studies that the strength of sensory-perceptual details can be either considered as one characteristic [27] or classified into visual and non-visual information [22,29]. Therefore, it is prudent to generate different items for visual and non-visual information of addiction memory intensity and to test the factor structure of the AMIS (i.e., whether these two components represent different constructs).

For generating items unique to measuring the intensity of addiction memory, in-depth interviews were conducted with twelve illicit drug users with substance use disorders. The respondents were asked to recall and describe their experiences of using drugs, and then to describe their subjective experience during retrieval of the memories of using drugs. The interview transcripts were reviewed, coded, and classified into the themes of visual and non-visual information via a thematic framework analysis. Forty-four items were then initially generated based on the abovementioned instruments and interviews.

Five psychologists with professional experience in mental health and addiction treatment were invited to a consultation meeting to review the preliminary instrument. Twenty illicit drug users in the drug rehabilitation centers were then interviewed about the understandability and acceptability of the scale and each of the items. Based on the feedback from the psychologists and illicit drug users, items that were ambiguous, repetitive, not understood, or not acceptable were either revised or removed, yielding a 20-item draft of the AMIS. The AMIS draft was piloted in a sample of 345 illicit drug users to evaluate the appropriateness of the items further (see Study-1). The results of Study-1 were used for item selection. After the item selection, a final version of the AMIS was developed, consisting of nine items.

In the present study, the AMIS was specifically developed to measure the intensity of addiction memory in illicit drug users. Given the fact that most of the illicit drug users in China have a low education level, we agree with the previous studies [34,35,36] that suggest that a fully labelled five-point Likert scale may improve the respondents’ ability to discriminate among categories and reduce response bias, without lowering the reliability of the instrument. Therefore, regarding the response options, five-point response categories were labelled (1 = strongly disagree, 2 = disagree, 3 = unsure, 4 = agree, 5 = strongly agree) and used on all the items. The total AMIS score can be obtained by computing the mean of the items; thus, the range of possible scores is from 1 to 5.

### 2.2. Participants

Two studies were conducted from January 2015 to March 2018 at drug rehabilitation centers in China. Participants were illicit drug users (substance use disorders as diagnosed by the DSM-IV) with the age of at least 18 years old. Illicit drug users who had difficulty in answering the survey as a result of illiteracy, withdrawal symptoms, cognitive disorders, or other psychiatric disorders were excluded from the studies. The study protocols were approved by the Institutional Review Board of the School of Public Health, Tongji Medical College, Huazhong University of Science and Technology (approval file number: [2015]#15). Each participant in the studies provided signed informed consent.

#### 2.2.1. Participants in Study-1

For item analysis, Study-1 was conducted at two drug rehabilitation centers in the city of Wuhan, China. Based on the lists provided by the rehabilitation centers, the illicit drug users were randomly selected by the principal researcher (Z.-Z.W.) using a list of computer-generated random numbers. Ultimately, a total of 345 participants were selected.

#### 2.2.2. Participants in Study-2

For initial validation of the AMIS, Study-2 was conducted at six drug rehabilitation centers in two cities (Wuhan and Zhongshan) in China. To avoid repetition, we excluded the individuals who participated in Study-1. Finally, a total of 1550 participants were recruited at the rehabilitation centers.

### 2.3. Measurements for Testing Concurrent Validity

In Study-2, the Obsessive Compulsive Drug Use Scale (OCDUS) [37] and a craving visual analogue scale (VAS) were used for concurrent validation since previous studies have indicated that addiction memory is related to craving [38,39,40,41]. Given that individuals who experienced a longer term of drug use may undergo more repeated exposures and thus strengthen their intensity of memory [42], the participants’ duration of illicit drug use was also used for concurrent validation.

#### 2.3.1. Obsessive Compulsive Drug Use Scale (OCDUS)

The OCDUS was used to measure general craving in the past week. The Chinese version of OCDUS is a 12-item questionnaire and uses a three-factor structure: “interference of drugs,” “frequency of craving,” and “control of drugs” [37].

#### 2.3.2. Visual Analogue Scale (VAS)

The VAS was used to measure instant craving. The VAS is a 10-cm line with “not at all” on the left and “extremely” on the right. Participants were asked to rate their craving for drugs at present on the VAS.

#### 2.3.3. Duration of Illicit Drug Use

The participants were asked to answer a single question adapted from the Chinese version of Addiction Severity Index-V [17] to report their duration of illicit drug use (“How many years in your life have you regularly used the illicit drugs (e.g., heroin, amphetamines, ketamine, cannabis, cocaine, or more than one substance)?”).

### 2.4. Procedure

The 20-item draft of the AMIS was administered in Study-1. Before answering the scale, participants were instructed to recall memories about their experiences of drug use (“Please recall your experiences of using drugs. Please try your best to recall the experiences in detail, for example, when and where it happened, whom you were with, and what you felt then.”). In Study-2, the nine-item AMIS was applied. Participants were given the instruction mentioned above and then completed the scale. Subsequently, they were asked to answer the OCDUS and the VAS. To avoid leaving the participants in a vulnerable state, they were given relaxation techniques after finishing the surveys.

### 2.5. Data Analysis

The samples available for data analysis numbered 343 in Study-1 and 1420 in Study-2. In Study-1, two participants were unwilling to answer the survey and did not provide any information. Thus, they were excluded from the study. In Study-2, one-hundred-and-thirty questionnaires with missing items were excluded from data analysis. The characteristics of participants who were excluded were not significantly different from those included in the data analysis (Supplementary materials, Table S1). All statistical analyses were performed in SAS 9.4 (SAS Institute Inc., Cary, NC, USA) [43], and the significance level was set at *α* = 0.05 (two-tailed probability).

#### 2.5.1. Study-1: Item Analysis

The critical ratio, item-total correlation, factor loading, and coefficient of stability were calculated for each item. Regarding the coefficient of stability, thirty of the participants were randomly selected using a list of computer-generated random numbers. They were retested two weeks after the first test. The results of the item analysis were used comprehensively for item selection. Items that meet two or more deletion criteria were removed [44]. The deletion criteria were as follows [45]: the critical ratio <3.00, the item-total correlation coefficient <0.40, factor loading <0.45, and coefficient of stability <0.50.

#### 2.5.2. Study-2: Initial Validation of the AMIS

For cross-validation, both exploratory factor analysis (EFA) and confirmatory factor analysis (CFA) were used to validate the factor structure of the AMIS. The sample data were randomly divided into two parts before the factor analyses were conducted. One subset was for EFA (*n* = 710), and the other was for CFA (*n* = 710).

The principal component analysis combined with oblique (direct oblimin) rotation was performed in EFA. The eigenvalue and the scree plot were used to assist in retaining the number of components.

The maximum likelihood estimation was conducted in CFA. The model chi-square statistic, comparative fit index (CFI), standardized root mean square residual (SRMR), root mean square error of approximation (RMSEA), and non-normed fit index (NNFI) were reported. The chi-square statistic was not used as a viable fit index because of its excessive sensitivity to sample size [46,47]. Thereby the acceptable fit-index criteria used in CFA were as follows [46,47]: CFI > 0.90, SRMR < 0.08, RMSEA < 0.10, and NNFI > 0.90. Additionally, the expected cross-validation index (ECVI) was tested to assist model comparison. The model with a lower ECVI value was considered to be better-fitting [46,47].

The impact of gender or primary illicit drug of use on the measurement invariance was tested in a series of multi-group CFAs. The measurement invariance could be justified if [48,49]: the overall fit of the model was acceptable, and the differences in CFI and NNFI between the constrained and unconstrained model were <0.01 and <0.05 respectively. Of the participants recruited, only 1.1% were ketamine users (see Table 1). Due to the insufficient sample size of this subgroup, the ketamine users were excluded from the multi-group CFA models when comparing different primary drugs of use. 

The inter-factor correlation and the concurrent validity of the AMIS were estimated using the Pearson correlation coefficient, using a full sample (*n* = 1420). When assessing the correlations between the AMIS and the VAS, the VAS scores were found to follow a non-normal distribution; thus, Spearman correlation coefficients were calculated instead. 

To evaluate whether the AMIS could discriminate between the high and moderate-to-low levels of craving, we performed a series of independent-samples *t*-tests. According to the previous study [50], the participants who scored >5 on the VAS were considered to have a high level of instant desire for drugs. Of the OCDUS, the following cut-off values indicate the high level of related contents: “interference of drugs” >15, “frequency of craving” >15, and “control of drugs” >6 [37].

Cronbach’s *α* coefficient was computed to evaluate the internal consistency of the AMIS in a full sample (*n* = 1420). Test-retest reliability of the AMIS was obtained from a retest conducted in a subset of sixty participants. The participants were retested two weeks after their first test. The test-retest reliability was assessed using the Pearson correlation coefficient.

## 3. Results

### 3.1. Characteristics of the Participants

The characteristics of the participants in our study are shown in Table 1.

### 3.2. Item Analysis

The results of the item analysis are shown in Table 2. Three items showed an item-total correlation coefficient of <0.40, eleven items showed a factor loading of <0.40, and ten items showed a coefficient of stability of <0.50. Based on the abovementioned criteria for item selection, nine items were ultimately retained in the AMIS.

### 3.3. Exploratory Factor Analysis (EFA)

**Figure 1:**
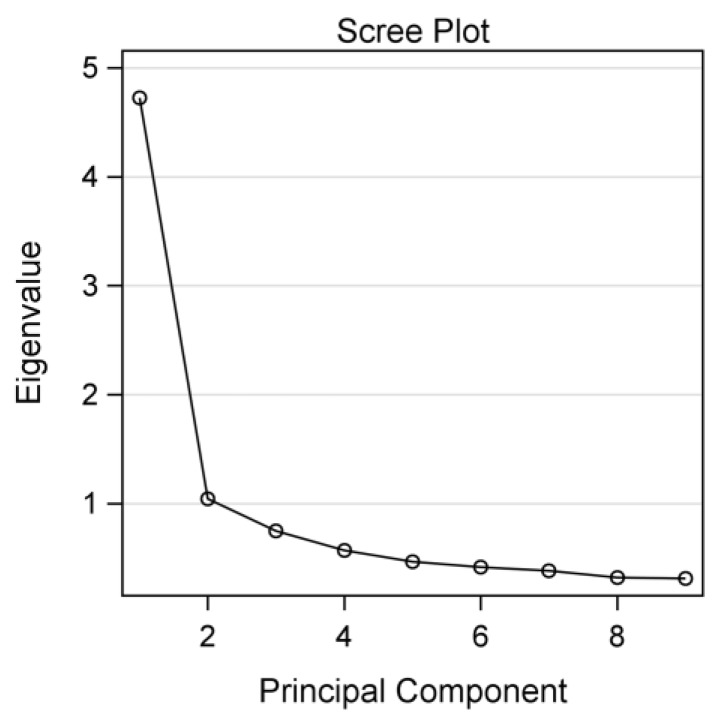
Scree plot based on the principal component analysis (*n* = 710).

Table 3 displays the factor structure of the AMIS. The Kaiser–Meyer–Olkin measure of sampling adequacy (0.90) and Bartlett’s test of sphericity (*χ*^2^ = 2910.00, *p* < 0.001) indicated a suitable correlation matrix for factor analysis. The pattern matrix showed a two-factor structure with each eigenvalue >1 (4.73 and 1.04). The scree plot also suggested this factor solution (Figure 1).

The two-factor solution of the AMIS accounted for 64.11% of the total variance. Factor-1, labelled Visual Clarity, had shown greatest loadings on six items and measured the intensity of one’s visual information when retrieving the memories of drug use. Factor-2, labelled Other Sensory Intensity, had shown greatest loadings on three items and evaluated the intensity of one’s non-visual sensations and feelings when recalling the experiences of using drugs.

Item-15 was suspected to be a cross-loading item (the difference in factor loading was 0.22, slightly over 0.20). Thus, we tried to delete this item and see how the factor solution and internal consistency were affected. After the factor analysis and sensitivity analysis (Supplementary materials, Table S2), we found that removing this item hardly changed the two-factor structure, but would reduce the internal consistency of the scale. Therefore, item-15 was retained in the AMIS.

### 3.4. Confirmatory Factor Analysis (CFA)

The two-factor structure of the AMIS from EFA provided an acceptable fit for the other subset of sample data (see Table 4, Model 1). An alternative one-factor model was also constructed to assess the intensity of sensory-perceptual information. All items in this model served as indicators of one single factor. The one-factor model showed an acceptable-to-poor fit to the sample data, with the RMSEA value failed to meet its fit-index criterion (see Table 4, Model 2). Due to the lower ECVI value, the two-factor solution was favored.

All of the model-fit indexes of the two-factor multi-group models met the acceptance criteria (see Table 4, Model 3 and Model 4). When constraining all parameters to be equal across groups, the differences between the constrained and unconstrained models showed ΔCFIs of <0.01 and ΔNNFIs of <0.05 (across gender: ΔCFI = −0.001, ΔNNFI = 0.007; across primary drug of use: ΔCFI = 0.000, ΔNNFI = 0.006). Also, no significant difference in model fits was observed between the constrained and unconstrained models (across gender: Δ*χ*^2^ (7) = 10.71, *p* = 0.152; across primary drug of use: Δ*χ*^2^ (7) = 5.57, *p* = 0.591). These results suggest measurement invariance across the groups.

### 3.5. Inter-Factor Correlation and Concurrent Validity

There was a moderate, significant correlation between the two factors of the AMIS (*r* = 0.65, *p* < 0.001). The correlations between the AMIS and other measures are shown in Table 5. Significant correlations were found between OCDUS scores, VAS scores, duration of illicit drug use, and AMIS scores.

### 3.6. Discriminant Validity

The *t*-test results showed that, compared to the participants with moderate-to-low levels of craving, those with high levels of craving reported significantly higher scores on the AMIS (Table 6), indicating that the AMIS can discriminate between the high and moderate-to-low levels of craving.

### 3.7. Reliability

Respectively, Cronbach’s *α* coefficients for the total, Visual Clarity, and Other Sensory Intensity scores on the AMIS were 0.89, 0.88, and 0.72, suggesting a good internal consistency in the scale.

Results from the retest showed that, between the test and the retest, the Pearson correlation coefficients for the AMIS and its two factors were good (Total: 0.80 (*p* < 0.01); Visual Clarity: 0.75 (*p* < 0.01); Other Sensory Intensity: 0.72 (*p* < 0.01)).

## 4. Discussion

This study develops and initially validates a new measure to assess the intensity of addiction memory in illicit drug users. The final version of the AMIS consists of nine items and shows a robust two-factor structure. Results of the multi-group CFA suggest measurement invariance of the two-factor structure across groups of different genders and different primary drugs of use. The moderate inter-factor correlation further confirms that the internal structure of the AMIS is reasonable. The validity of the AMIS is supported by other findings that there are significant correlations between the AMIS and other measures of craving and that the AMIS can discriminate between different levels of craving. Additionally, the AMIS and its two factors show good internal consistency and test-retest reliability. These results support the AMIS as a valid and reliable tool for measuring the intensity of addiction memory in illicit drug users.

The AMIS was initially developed to assess the intensity of sensory-perceptual information in addiction memory of the illicit drug users. Two phenomenological characteristics (Visual Clarity and Other Sensory Intensity) were identified in EFA. It is notable that the contents of these two factors are consistent with the theoretical dimensions constructed from our assumption. Thus, it is easy to interpret the factors clinically, as follows: Visual Clarity refers to the intensity of illicit drug users’ visual information when retrieving the memories of drug use, while Other Sensory Intensity refers to the strength of their non-visual sensations and feelings. Moreover, Visual Clarity explained 52.51% of the total variance, which was higher than Other Sensory Intensity (11.60%). That is, the clarity of visual information accounts for a percentage of variance in the intensity of addiction memory that is greater than other sensory details. Similar to other studies that demonstrate that visual information predominates in the episodic memories [32,33], the results of our study support the significance of visual clarity in the intensity of addiction memory.

Although researchers have differentiated visual clarity from other sensory intensity [22,29], the two components can also be considered as one characteristic which reveals the strength of all sensory-perceptual details [27]. Thus, we compared the two-factor model to an alternative one-factor model. The two-factor solution showed an acceptable fit to the sample data and, due to its lower ECVI value, was considered to fit the data better. Furthermore, it is indicated in the cross-validation that the two-factor structure would be more stable. Therefore, the two-factor solution was preferred. Based on the results of factor analyses and inter-factor correlation analysis, the Visual Clarity and Other Sensory Intensity factors can be considered two related but distinct characteristics of addiction memory intensity.

Regarding the concurrent validity, scores of the AMIS and its factors showed significant correlations with the measures of craving, which is consistent with previous studies, suggesting that addiction memory is related to craving [38,39,40,41]. Although the results provide support for the validation of the AMIS, it should also be noted that there were significant but only modest correlations between the AMIS and the measures of craving. This may be due to the fact that addiction memory is long-lasting and resistant to being extinguished, whereas craving is considered to be a transient, fluctuant state that is sensitive to environmental influences [51,52,53]. In other words, individuals who retrieve vivid details from their memories of using drugs may, if there are other influences, report a low desire for drug use. In our study, the participants did rate extremely low scores on both the measures of craving (Supplementary materials, Table S3), especially the VAS (over one-third of the participants scored 0). Similar situations can be found in other studies. For example, Anton et al. [54] found that the high ratings on craving diminished when the patients were either hospitalized or successfully participated in treatment. Since the participants in our study were undergoing rehabilitation, it might be the engagement in treatment (which may result in a high self-efficacy or mastery of coping skills) that assisted them to prevent the escalation of desire for drug use. That is, it seems the interaction between craving and environmental influences that interfere with the association between the intensity of addiction memory and the level of craving. Significant correlations were also found between the AMIS scores and the duration of illicit drug use. However, the associations were quite modest, which may suggest that the participants’ duration of illicit drug use, as measured in our study, is not particularly related to the intensity of addiction memory. It is possible that there may be other factors, such as the strength of the unconditioned stimulus [9], that moderate the associations between the intensity of addiction memory and the duration of illicit drug use. For example, a higher level of frequency or dose of drug use may elicit a stronger memory of addiction, which means the intensity of addiction memory may vary in degrees of frequency or dose of drug use but not merely depend on the duration of drug use. Future studies are needed to collect the participants’ full history of drug use and to explore how the drug use history may affect the intensity of addiction memory.

Our study has several limitations that should be considered. First, the participants in our study were hospitalized in the drug rehabilitation centers and could not provide any information on current illicit drug use. Therefore, only craving was measured to evaluate the concurrent validity of AMIS. Second, although we have taken considerable effort to administer the AMIS to a diverse sample, the final version of the scale is a result of the sample used in the current study. Like other scales, the AMIS focused on a specific population (illicit drug users). Given the commonalities across addictions, the items of the AMIS may be adapted for patients with tobacco dependence, alcohol dependence, and so on. However, based on the current study, it is prudent not to draw an over-interpreted conclusion. Whether the AMIS can be decontextualized to produce such a broader application, is unknown. Moreover, although the measurement invariance indicated an absence of difference across primary illicit drug of use, the samples of illicit drug users in this study were primarily using heroin and amphetamine-type stimulants but no other illicit drugs such as cocaine and cannabis; the sample data was not collected from adolescents or non-Chinese speakers either. Thus, it is unknown whether the results of this study can be generalized to other populations of illicit drug users. Finally, although this study initially validates the utility of the AMIS, the scale should further be validated in reconsolidation-based interventions to see whether it can predict responses to the interventions. Also, since the participants were not followed-up, other studies are required to determine the predictive validity of the AMIS.

Regardless of the limitations, this study presents a promising measure to assess the intensity of addiction memory in illicit drug users. The AMIS allows the clinicians and researchers to gather information regarding the strength of addiction memory from a multidimensional perspective. Measurement invariance of the factor structure indicates that the AMIS can be reliably utilized to evaluate the intensity of addiction memory in, at least, heroin and amphetamine-type stimulant users, and thus enable comparison of the memory intensity across illicit drug users. Furthermore, the AMIS may tailor interventions based on responses to the scale, especially the interventions based on memory reconsolidation, although future studies are needed to validate the responsiveness of the AMIS. Finally, the brief nature of the AMIS makes it easy to administer, so that the clinicians and researchers can use the instrument to measure the intensity of addiction memory efficiently.

## 5. Conclusions

In conclusion, the present study is the first to develop a scale for measuring the intensity of addiction memory in illicit drug users. The current evidence suggests that the AMIS is a reliable and valid tool with the advantages of being brief and easily applied, and has the potential to be useful in future clinical research.

## Figures and Tables

**Table 1:** Characteristics of the participants.

Characteristics	Study-1 (*n* = 343)	Study-2 (*n* = 1420)
Gender (%)		
Male	49.3	50.4
Female	50.7	49.6
Mean age (SD)	35.6 (8.5)	38.4 (9.2)
Education (%)		
Primary school or below	12.5	18.4
Junior high school	55.7	51.1
Senior high school	25.7	24.9
Junior college or above	6.1	5.6
Marital status (%)		
Single	41.1	34.8
Premarital cohabitation	2.3	2.4
Married	38.8	36.0
Divorced or widowed	17.8	26.8
Primary illicit drug of use (%)		
ATS	47.8	43.9
Heroin	26.5	40.1
Ketamine	3.5	1.1
Polydrug use	22.2	14.9
Mean years of illicit drug use (SD)	7.4 (5.9)	10.4 (6.8)

Note: ATS = amphetamine-type stimulants; SD = standard deviation.

**Table 2:** Item analysis of the draft Addiction Memory Intensity Scale (AMIS; *n* = 343).

Items of the AMIS Draft	Mean (SD)	Critical Ratio	Item-Total Correlation	Factor Loading	Coefficient of Stability	Items Retained
Item-1: recall the images clearly	2.94 (1.19)	6.80	0.46	0.74	0.78	Yes
Item-2: recall the images coherently	2.97 (1.10)	8.84	0.53	0.79	0.71	Yes
Item-3: the sensations and feelings spring to my mind	2.94 (1.17)	6.35	0.45	0.69	0.77	Yes
Item-4: feel what I felt then	2.64 (1.09)	5.82	0.42	0.65	0.72	Yes
Item-5: recall the images as an outsider	3.10 (1.06)	5.52	0.32 *	−0.08 *	−0.06 *	
Item-6: recall the images in detail	3.07 (1.10)	8.94	0.54	0.80	0.81	Yes
Item-7: recall a certain instance at a particular time and place	3.13 (1.11)	6.77	0.45	0.71	0.83	Yes
Item-8: recall the images easily	3.00 (1.12)	9.55	0.57	0.78	0.75	Yes
Item-9: the sensations and feelings are intense	2.31 (1.12)	6.85	0.43	0.51	0.74	Yes
Item-10: the images are far away from me	2.89 (1.09)	4.47	0.34 *	−0.11 *	−0.05 *	
Item-11: the images are dim	2.90 (1.08)	8.41	0.50	0.10 *	0.08 *	
Item-12: recall a merging of many images	3.07 (1.07)	3.56	0.23 *	−0.20 *	0.57	
Item-13: had to search my memory to recall the images	2.99 (1.10)	7.33	0.41	−0.07 *	0.42 *	
Item-14: the evoked physical reactions are weak	2.67 (1.15)	7.77	0.45	−0.02 *	0.19 *	
Item-15: the images are deeply rooted in my mind	2.84 (1.07)	6.03	0.42	0.61	0.65	Yes
Item-16: the images are sketchy	2.94 (1.01)	7.66	0.47	0.03 *	0.31 *	
Item-17: recall the images in pieces	3.05 (1.17)	11.93	0.55	0.08 *	0.41 *	
Item-18: had to search my memory to recall the feelings	3.08 (1.11)	13.61	0.59	0.19 *	0.11 *	
Item-19: relive the experiences without any sensations and feelings	2.76 (1.16)	9.91	0.52	0.08 *	0.38 *	
Item-20: the sensations and feelings are impressive	2.82 (1.17)	14.75	0.65	0.28 *	0.30 *	

Note: * Items that meet the deletion criteria; AMIS = Addiction Memory Intensity Scale; SD = standard deviation.

**Table 3:** Exploratory factor analysis of the Addiction Memory Intensity Scale (AMIS; *n* = 710) ^1^.

Items of the AMIS ^2^	Factor 1	Factor 2
**Factor 1: Visual Clarity (eigenvalue = 4.73, % variance = 52.51)**		
Item-7: I can recall a certain instance of using drugs at a particular time and place.	0.85	−0.10
Item-6: I can recall the images of using drugs in detail.	0.82	0.05
Item-1: I can recall the images of using drugs clearly.	0.81	−0.04
Item-2: When I recall the images of using drugs, they emerge coherently.	0.80	0.02
Item-8: I can recall the images of using drugs easily.	0.75	0.06
Item-15: When I recall the images of using drugs, I find that they are deeply rooted in my mind.	0.53	0.31
**Factor 2: Other Sensory Intensity (eigenvalue = 1.04, % variance = 11.60)**		
Item-9: When I recall my experiences of using drugs, the sensations and feelings are intense.	−0.16	0.91
Item-4: When I recall my experiences of using drugs, I can feel what I felt then.	0.20	0.72
Item-3: When the drugs or drug-related cues are present, the sensations and feelings of using drugs spring to my mind.	0.26	0.59

Note: ^1^ The pattern matrix was presented; factors were extracted by principal component analysis and were rotated by oblique (direct oblimin) rotation. ^2^ Numbering reflects the order of items on the original scale. The AMIS was originally developed in Chinese, and the English version presented here was translated only for the publication of our study. AMIS = Addiction Memory Intensity Scale.

**Table 4:** Confirmatory factor analysis of the Addiction Memory Intensity Scale (AMIS; *n* = 710).

Model	*χ*^2^ (*df*)	CFI	SRMR	RMSEA	NNFI	ECVI
1. The hypothesized two-factor model	175.88 (25) *	0.95	0.05	0.09	0.93	0.31
2. The alternative one-factor model	218.25 (26) *	0.94	0.05	0.10	0.92	0.36
3. Multi-group model comparing gender	207.28 (50) *	0.95	0.04	0.07	0.93	
4. Multi-group model comparing the primary drug of use	296.46 (95) *	0.94	0.06	0.06	0.93	

Note: * *p* < 0.01; AMIS = Addiction Memory Intensity Scale; CFI = comparative fit index; SRMR = standardized root mean square residual; RMSEA = root mean square error of approximation; NNFI = non-normed fit index; ECVI = expected cross-validation index.

**Table 5:** Correlations between the Addiction Memory Intensity Scale and other measures (*n* = 1420).

Measure	Addiction Memory Intensity Scale		
	Total	Visual Clarity	Other Sensory Intensity
OCDUS			
Interference of drugs	0.25 *	0.20 *	0.27 *
Frequency of craving	0.42 *	0.39 *	0.38 *
Control of drugs	0.24 *	0.21 *	0.23 *
VAS	0.20 *	0.14 *	0.25 *
Duration of illicit drug use	0.08 *	0.07 *	0.09 *

Note: * *p* < 0.01. OCDUS = Obsessive Compulsive Drug Use Scale; VAS = Visual Analogue Scale.

**Table 6:** Discriminant validity of the Addiction Memory Intensity Scale (*n* = 1420).

Measure	Addiction Memory Intensity Scale (Mean (SD))		
	Total	Visual Clarity	Other Sensory Intensity
OCDUS			
Interference of drugs			
Moderate-to-low	3.3 (0.8)	3.4 (0.9)	3.0 (0.9)
High	4.1 (0.8)	4.2 (0.8)	4.0 (0.9)
	*t* = −11.58, *p* < 0.001	*t* = −10.10, *p* < 0.001	*t* = −11.38, *p* < 0.001
Frequency of craving			
Moderate-to-low	3.3 (0.8)	3.4 (0.9)	3.0 (0.9)
High	4.4 (0.4)	4.5 (0.5)	4.3 (0.6)
	*t* = −23.71, *p* < 0.001	*t* = −20.51, *p* < 0.001	*t* = −19.87, *p* < 0.001
Control of drugs			
Moderate-to-low	3.3 (0.8)	3.4 (0.9)	3.0 (0.9)
High	4.2 (0.7)	4.2 (0.8)	4.0 (0.9)
	*t* = −11.66, *p* < 0.001	*t* = −11.78, *p* < 0.001	*t* = −10.93, *p* < 0.001
VAS			
Moderate-to-low	3.3 (0.9)	3.4 (0.9)	3.0 (1.0)
High	3.6 (0.8)	3.6 (0.8)	3.5 (0.9)
	*t* = −5.10, *p* < 0.001	*t* = −3.80, *p* < 0.001	*t* = −6.98, *p* < 0.001

Note: OCDUS = Obsessive Compulsive Drug Use Scale; VAS = Visual Analogue Scale.

## Supplementary Materials

The following are available online at http://www.mdpi.com/2077-0383/7/12/467/s1. Table S1: Comparison of the participants’ characteristics in Study-2; Table S2: Factor loading and internal consistency of the Addiction Memory Intensity Scale if item-15 is deleted; Table S3: Descriptive data for measures in Study-2.
