# Supplementary material for: A New Measure for Assessing the Intensity of Addiction Memory in Illicit Drug Users: The Addiction Memory Intensity Scale
Source: J Clin Med. 2018 Nov 22;7(12):467. doi: 10.3390/jcm7120467 (PMC6306924; doi:10.3390/jcm7120467)
Supplement: Supplementary file 1 [file jcm-07-00467-s001.pdf]

Table S1. Comparison of the participants' characteristics in Study-2.

Characteristics	Participants Included in Study-2 (n = 1420)	Participants Excluded from Study-2 (n = 130)	χ^2 or t-Value	p-Value
Gender (%)				
Male	50.4	45.4	1.21	0.272
Female	49.6	54.6		
Mean age (SD)	38.4 (9.2)	38.6 (6.0) [1]	0.32	0.753
Education (%)				
Primary school or below	18.4	17.7	4.30	0.231
Junior high school	51.1	54.6		
Senior high school	24.9	22.3		
Junior college or above	5.6	1.5		
Missing values	-	3.8		
Marital status (%)				
Single	34.8	32.3	1.44	0.697
Premarital cohabitation	2.4	1.5		
Married	36.0	39.2		
Divorced or widowed	26.8	23.1		
Missing values	-	3.8		
Primary illicit drug of use (%)				
ATS	43.9	43.1	3.00	0.393
Heroin	40.1	35.4		
Ketamine	1.1	1.5		
Polydrug use	14.9	20.0		
Mean years of illicit drug use (SD)	10.4 (6.8)	9.4 (6.4) [2]	1.54	0.123

[1] Missing information in 8 participants. [2] Missing information in 11 participants. Note: ATS = amphetamine-type stimulants; SD = standard deviation.

Table S2. Factor loading and internal consistency of the Addiction Memory Intensity Scale if Item-15 is deleted (*n* = 710).

Items	Factor-1: Visual Clarity (Eigenvalue = 4.26, %Variance = 53.21)	Factor-2: Other Sensory Intensity (Eigenvalue = 1.04, %Variance = 13.03)	Cronbach's α for the Total Scale If Item Is Deleted [1]	Cronbach's α for Factor-1 If Item Is Deleted [2]
Item-1	0.81	0.02	0.869	0.861
Item-2	0.79	0.04	0.865	0.854
Item-3	0.26	0.62	0.874	
Item-4	0.20	0.74	0.869	
Item-6	0.81	0.07	0.861	0.846
Item-7	0.84	0.09	0.870	0.859
Item-8	0.75	0.07	0.867	0.858
Item-9	0.16	0.90	0.888	
Item-15			0.869	0.869

[1] Cronbach's α coefficients for the total scale was 0.883. [2] Cronbach's α coefficients for Factor-1 was 0.879. Note: Please refer to Table 3 for detailed information of the items. The pattern matrix was presented. Factors were extracted by principal component analysis and were rotated by oblique (direct oblimin) rotation.

Table S3. Descriptive data for measures in Study-2 (n = 1420).

Measure	Mean	Median	SD	Range
AMIS				
Total	3.35	3.44	0.85	1–5
Visual clarity	3.47	3.50	0.90	1–5
Other sensory intensity	3.10	3.00	0.97	1–5
OCDUS				
Interference of drugs	7.80	7.00	4.97	0–20
Frequency of craving	7.40	7.00	4.76	0–20
Control of drugs	3.77	4.00	1.86	0–8
VAS [1]	2.84	2.00	3.13	0–10

[1] The VAS scores were found to follow a non-normal distribution; thus, Median should be used instead of Mean. Note: AMIS = Addiction Memory Intensity Scale; OCDUS = Obsessive Compulsive Drug Use Scale; VAS = Visual Analogue Scale; SD = standard deviation.
